# HPMA polymers as functional excipients in dermal nanoformulations of imiquimod

**DOI:** 10.1016/j.ijpx.2026.100486

**Published:** 2026-01-08

**Authors:** Eliška Kurfiřtová, Stanislav Chvíla, Nikola Strnádková, Vendula Janoušková, Petr Chytil, Tomáš Etrych, Jarmila Zbytovská

**Affiliations:** a University of Chemistry and Technology Prague, Faculty of Chemical Technology, Department of Organic Technology, Technická 5, 166 28 Prague, Czech Republic; b University of Chemistry and Technology Prague, Faculty of Chemical Engineering, Department of Chemical Engineering, Technická 5, 166 28 Prague, Czech Republic; c Institute of Macromolecular Chemistry, Czech Academy of Sciences, Heyrovského nám. 2, Prague 6 162 06, Czech Republic

**Keywords:** HPMA polymers, Skin penetration enhancers, Imiquimod, Nanocrystals, Nanoemulsion, Dermal delivery

## Abstract

A key challenge in topical drug delivery is the inherently low bioavailability of many active compounds within skin tissue. Here, we present the first comprehensive study investigating the impact of biocompatible hydrophilic polymers based on *N*-(2-hydroxypropyl)methacrylamide (p(HPMA)) on skin barrier properties and its potential to enhance drug permeation. Using imiquimod (IMQ), a model compound known for its poor dermal delivery, we demonstrate that p(HPMA) can significantly influence transport across the skin. To enhance the dermal delivery of IMQ, we investigated three p(HPMA) polymers of varying molecular sizes (5, 20, 80 kg/mol) with very low dispersity. Our initial focus was on the p(HPMA) interaction with the skin barrier, specifically within the *stratum corneum* (SC), which was studied by confocal microscopy. Results revealed that p(HPMA) can penetrate into deeper skin layers, with this ability inversely correlated with their molecular weight. FTIR analysis confirmed that the polymers increase SC hydration without disrupting lipid organization. As demonstrated by the *ex vivo* skin permeation study, the smallest p(HPMA) polymer (5 kg/mol) produced the strongest enhancement effect on IMQ delivery into skin tissue. Relative to p(HPMA)-free controls, IMQ accumulation increased by 90% from the conventional suspension and by 10% and 50% from the nanoemulsion and nanocrystal formulations, respectively. These findings substantiate the role of p(HPMA) as an effective skin-penetration enhancer and support its further investigation for optimizing topical drug-delivery systems.

## Highlights

• HPMA polymers penetrate skin barrier and accumulate in tissue; smaller molecules penetrate more efficiently.
• Although HPMA polymers do not interact with skin barrier lipids, they influence hydration of its protein components.
• HPMA polymers act as potent skin-penetration enhancers for IMQ across both traditional and nanoparticulate formulations.

## Graphical abstract

**Figure:**
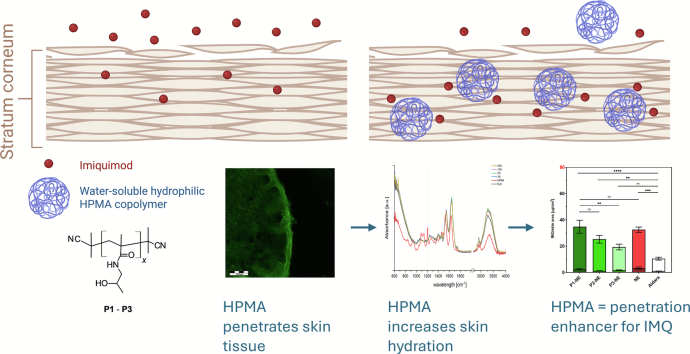

## 1 Introduction

In dermal drug delivery, effectiveness largely depends on the drug's ability to traverse the *stratum corneum* (SC), the skin's outermost and most significant barrier, and reach the deeper layers where it can exert its therapeutic effect (Gorzelanny et al., 2020; Roger et al., 2019). To overcome the skin barrier, a variety of strategies have been employed, which can be broadly categorized as either passive or active methods. While active methods rely on external energy input, passive methods enhance skin permeability through interactions between formulation components and the skin barrier, as well as within the formulation itself. These interactions result in a temporary increase in skin permeability to active substances (Chaturvedi and Garg, 2021; Vitorino et al., 2014). Several fundamental strategies are currently employed to optimize the penetration of active substances into the skin, primarily by increasing their thermodynamic activity within the formulation or enhancing their solubility in the SC.

A traditional strategy to enhance skin penetration relies on the use of penetration enhancers, which can act through multiple mechanisms. Small molecules, such as alcohols, can improve drug solubility within the vehicle or directly in the lipid matrix of the SC. Amphiphilic compounds, such as Azone or oleic acid, interact with SC lipids, disrupting their ordered structure and increasing membrane fluidity, thereby facilitating drug permeation (Kováčik et al., 2020).

Recently, certain polymers have emerged as promising penetration enhancers due to their diverse mechanisms of action. For example, hyaluronic acid can traverse the SC, promote skin hydration, and improve the delivery of co-administered actives, while poly(amidoamine) (PAMAM) dendrimers have been shown to increase drug flux through the SC (Gökçe et al., 2021; Juhaščik et al., 2022; Venuganti et al., 2011; Zhu et al., 2020). Modern techniques for targeted dermal delivery often integrate these principles, with nanoparticle-based formulations (Kotla et al., 2017; Liu et al., 2023; Patzelt et al., 2017). Encapsulating actives into nanoparticles can enhance their thermodynamic activity, increase adhesion to the skin surface, and even form reservoirs in hair follicles, supporting sustained and localized delivery (Jain et al., 2011; Kalvodová et al., 2023).

Our research has previously focused on overcoming the limited dermal bioavailability of imiquimod (IMQ), used for, *e.g.* basal cell carcinoma or actinic keratosis (precancerous skin lesions). IMQ is known to enhance the immune response against infections and abnormal skin cells (Hanna et al., 2016). However, it exhibits poor skin penetration in conventional gels or creams (Al-Mayhay et al., 2019). Advanced nanocarrier systems, including nanocrystals (NC) and an oleic acid-based nanoemulsion (NE), were developed and demonstrated improved targeting of skin tissue (Petrová et al., 2023). Nanocrystals were also successfully integrated into hydrogel microneedle patches, illustrating the potential of combining nanoparticles with hydrophilic polymers for enhanced dermal delivery (Petrová et al., 2025).

Building on these findings, polymers based on *N*-(2-hydroxypropyl)methacrylamide (HPMA) represent an intriguing yet unexplored class of compounds as potential carriers for dermal penetration. They have shown considerable promise in biomedical applications due to their water solubility, biocompatibility, and lack of toxicity or immunogenicity (Chytil et al., 2018; Kopecek and Kopecková, 2010). When conjugated with drugs, HPMA-based systems can enable controlled release and targeted delivery to specific tissues or cells. Moreover, their structure can be precisely tuned using reversible addition–fragmentation chain-transfer (RAFT) polymerization (Scales et al., 2005) to achieve defined molecular weights and low dispersity. This can make them suitable candidates for skin delivery systems where particle size is a critical factor. Despite their proven advantages in targeted drug delivery (Chytil et al., 2021; Klepac et al., 2025; Lammers et al., 2007), the effect of HPMA polymers on the skin barrier and their potential to modulate its permeability remains unexplored.

The present study represents the first investigation of HPMA polymers (p(HPMA)) in dermal delivery. We synthesize three p(HPMA) polymers of varying molecular weights and evaluate their interaction with the skin barrier and their capacity to penetrate deeper skin layers, using fluorescently labelled variants. Using IMQ as a model active compound, we further assess the ability of p(HPMA) to enhance dermal transport from both conventional suspensions and nanoparticle systems. The aim is not to develop a final IMQ formulation, but rather to map the potential of p(HPMA) as a novel tool for modulating skin permeability and improving topical drug delivery.

## 2 Experimental methods

### 2.1 Materials

Polymer synthesis: 2,2′-azobisisobutyronitrile (AIBN), 2-cyanopropan-2-yl dithiobenzoate (CTA-DTB), methacryloyl chloride, *N*,*N*-diisopropylethylamine (DIPEA), dimethylsulfoxide (DMSO), *N*,*N*-dimethylacetamide (DMA), and 2-methylpropan-2-ol (*t*-BuOH) were purchased from Sigma-Aldrich (Czech Republic). The initiator 2,2′-azobis(4-methoxy-2,4-dimethylvaleronitrile) (*V*-70) was acquired from Fujifilm Wako Chemicals Europe (Germany), and the fluorescent dye DY-490 amino-derivative from Dyomics (Germany).

Dermal formulation and *ex vivo* experiments: IMQ was purchased from Cayman Chemical (Michigan, USA). Phospholipid GmbH (Germany) kindly provided Phospholipon® 90 G. Oleic acid, Tween® 80, methanol, acetonitrile, propylene glycol, gentamicin sulfate and phosphate-buffered saline (PBS; 10 mmol phosphate buffer, 2.7 mmol potassium chloride and 137 mmol sodium chloride, pH 7.4, at 25 °C) tablets were obtained from Merck KGaA (Germany). Aldara® (5 wt%) cream was purchased from MEDA AB (Sweden). All the chemicals were of analytical grade and used without further purification. Water was deionized, distilled, and filtered through a Millipore Q purification system.

### 2.2 P(HPMA) polymers preparation

#### 2.2.1 Synthesis of monomers and chain transfer agent

*N*-(2-hydroxypropyl)methacrylamide (p(HPMA)) and *N*-methacryloyl-*β*-alanine thiazolidine-2-thione (MA-AP-TT) were synthesized as described previously (Chytil et al., 2010; Šubr and Ulbrich, 2006). The chain transfer agent S-2-cyano-2-propyl-S`-ethyl trithiocarbonate (CTA-TTC) was prepared as described earlier (Koziolová et al., 2018).

#### 2.2.2 Synthesis of p(HPMA)

**Fig. 1:**
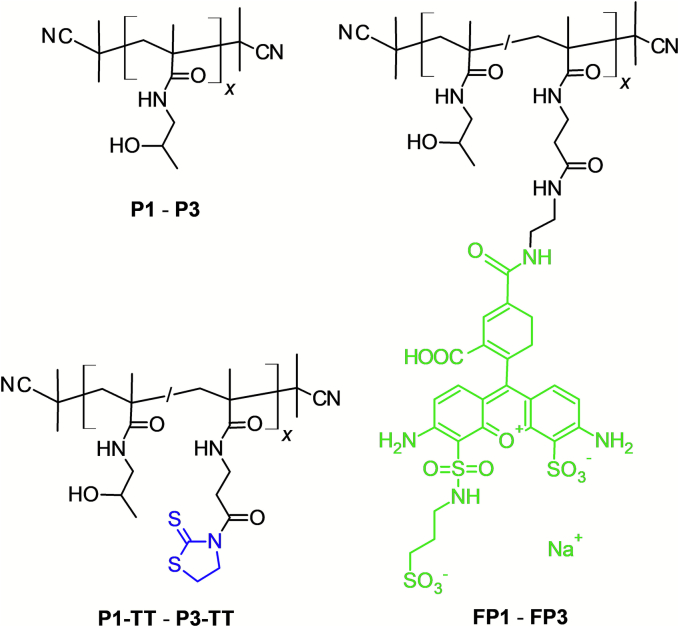
Schematic structures of the used pHPMA polymers. (TT groups are marked in blue and DY-490 in green.) (For interpretation of the references to colour in this figure legend, the reader is referred to the web version of this article.)

Polymers p(HPMA) (**P1** – **P3**) were synthesized by RAFT polymerization using *V*-70 as initiator and CTA-TTC or CTA-DTB as chain transfer agent followed by the polymer end-group removal *via* the reaction with an excess of AIBN according to (Perrier et al., 2005). The schematic structure of polymers is shown in Fig. 1.

Synthesis of polymer **P1**: HPMA was synthesized by RAFT polymerization using the molar ratio HPMA/CTA-TTC/V-70 70/2/1 in the mixture of *t*-butyl alcohol and DMSO 9/1 vol. at 0.8 M concentration. HPMA (1.00 g, 7.0 mmol) was dissolved in *t*-BuOH (7.9 mL) and mixed with a solution of V-70 (30.8 mg, 100 μmol), and CTA-TTC (41.0 mg, 200 μmol) in dry DMSO (0.9 mL). The reaction mixture was poured into a glass ampoule, bubbled with argon, and sealed. After 60 h in a thermostat-controlled water bath at 30 °C, the ampoule was cooled, and the reaction mixture was isolated by precipitation into 200 mL of acetone. After centrifugation, the crude polymer was purified by reprecipitation from methanol (9 mL) into ethyl acetate (200 mL). The polymer was filtered off and dried under vacuum. For the removal of the trithiocarbonate groups, the intermediate polymer and AIBN (20 wt% to polymer) were dissolved in DMA (10 wt% solution), poured into a glass ampoule, bubbled with argon, and sealed. After 3 h in a thermostat-controlled water bath at 80 °C, the solution was isolated and precipitated as reported above. The sample was dried under vacuum.

Synthesis of polymer **P2**: HPMA polymer was synthesized by RAFT polymerization using the molar ratio HPMA/CTA-DTB/*V*-70450/2/1 in the mixture of t-butyl alcohol and DMA 85/15 vol. at 0.7 M concentration. HPMA (1.00 g, 7.0 mmol) was dissolved in *t*-butylalcohol (8.5 mL) and mixed with a solution of V-70 (4.8 mg, 31 μmol), and CTA-DTB (6.9 mg, 16 μmol) in dry DMA (1.5 mL). The reaction mixture was poured into a glass ampoule, bubbled with argon, and sealed. The polymerization was carried out for 16 h in a thermostat-controlled water bath at 40 °C. The purification and removal of dithiobenzoate groups were the same as above for the removal of the trithiocarbonate groups.

Synthesis of polymer **P3**: HPMA polymer was synthesized by RAFT polymerization using the molar ratio HPMA/CTA-TTC/V-701400/2/1 in the mixture of t-butyl alcohol and DMSO 9/1 vol. at 0.5 M concentration. HPMA (1.00 g, 7.0 mmol) was dissolved in *t*-butyl alcohol (12.6 mL) and mixed with a solution of V-70 (1.5 mg, 5 μmol), and CTA-TTC (2.0 mg, 10 μmol) in dry DMSO (1.4 mL). The reaction mixture was poured into a glass ampoule, bubbled with argon, and sealed. Polymerization was carried out for 60 h in a thermostat-controlled water bath at 30 °C. The purification and removal of the dithiobenzoate groups were the same as above for the removal of the trithiocarbonate groups.

#### 2.2.3 Synthesis of fluorescently labelled polymers

The fluorescently labelled polymers (**FP1** – **FP3**) were prepared in two steps. First, statistical polymers poly(HPMA-*co*-MA-AP-TT) (**P1-TT** – **P3-TT**) were prepared by RAFT polymerization of HPMA and MA-AP-TT in the molar ratio 98/2. The reaction condition, purification, and polymer end-group removal were the same as described above for homopolymers **P1** – **P3**. The schematic structures of copolymers are shown in Fig. 1.

In the second step, thiazoline-2-thione (TT) groups of polymers **P1-TT** – **P3-TT** were used for the attachment of an amino-modified fluorescent dye DY-490 according to our previous work (Weiss et al., 2018). Each polymer precursor (50 mg) was dissol*v*ed in fresh methanol (400 μL) and mixed with a solution of DY-490 amino-derivative (1 mg) in 100 μL of fresh methanol under stirring. After 2 h, 2 μL of DIPEA was added. Then the reaction mixtures were kept under stirring overnight in the dark and purified using gel filtration on a Sephadex LH-20 column with methanol elution. The obtained polymers **FP1** – **FP3** were isolated by freeze-drying.

#### 2.2.4 Physicochemical characterisation of polymers

The weight-average molecular weight (*M*_w_) and dispersity (*Đ*) were determined by a Shimadzu high-performance liquid chromatography (HPLC) system equipped with the TSKgel Super SW3000 column (4.6 × 300 mm, 4 μm). An Optilab-rEX differential refractometer index (RI) (Wyatt Technology Co., USA), a multi-angle light scattering (MALS) (DAWN HELEOS II, Wyatt Technology Co., USA), and an SPD-M20A photodiode array (Shimadzu, Japan) detectors were used for analysis. Methanol/0.3 M sodium acetate buffer, pH 6.5 (8/2, *v/v*), was used as a mobile phase at a 0.5 mL·min^−1^ flow rate. ASTRA VI software was used to calculate *M*_w_, and *Ð*. The RI detector enabled direct determination of the polymers' refractive increment (*dn/dc*), and the solvent refractive index showed that 100% of the injected sample was recovered from the column.

The hydrodynamic radius (*R*_h_) of polymers was measured by dynamic light scattering (DLS) using a Nano-ZS instrument (ZEN3600, Malvern, UK) at *λ* = 632.8 nm and *θ* = 173°. Samples were prepared in PBS (pH 7.4) at 2 mg/mL using a 0.45 μm polyvinylidene fluoride (PVDF) filter. The values were determined as a mean of at least five independent measurements, and all data were evaluated using DTS(Nano) software.

The molar content of TT groups was determined by UV-VIS spectrophotometry using a Specord 205 ST (Analytic Jena AG, Germany) as described in our previous work (Šubr and Ulbrich, 2006). The molar absorption coefficient was *ε*(TT) = 10,300 L mol^−1^ cm^−1^ in methanol (*λ*_max_ = 305 nm). The samples were measured against methanol as a reference.

The content of the dye DY-490 was determined by UV-VIS spectrophotometry using a Specord 205 ST. Molar absorption coefficient ε_491_ = 73,000 L mol^−1^ cm^−1^ (PBS) was used to calculate the dye content according to the manufacturer's protocol. The samples were measured against PBS as a reference.

### 2.3 Nanoparticles preparation

#### 2.3.1 Nanoemulsion

Nanoemulsion (NE) was prepared according to the standard procedure published recently (Petrová et al., 2023). IMQ (2 wt%) was added together with Phospholipon® 90G (1.5 wt%) and Tween® 80 (3.5 wt%) to oleic acid (10 wt%), the mixture was heated to 60 °C until the solution became homogeneous. Subsequently, oleic acid with the dissolved components was mixed with distilled water (83 wt%) and homogenized by a high-shear homogenizer (Ultra-Turrax®, IKA, Germany) for 15 min followed by high-pressure homogenization (20 cycles; Emulsiflex C5, Avestin, Germany).

#### 2.3.2 Nanocrystals

Nanocrystals (NC, 2 wt% in final formulation) were prepared *via* small-scale wet-stirred medium milling (Petrová et al., 2023). For each run, 60 mg of IMQ, along with stabilizer (15 mg of Tween 80) pre-dissolved in 1 mL of deionized water, were placed into a 25 mL amber glass vial. The milling mediator was 5 g of ZrO milling beads (d = 0.4–0.5 mm). A 9 × 25 mm PTFE cross stirrer was used for agitation at 600 rpm. The milling was performed at room temperature for 24 h. Subsequently, the suspension was diluted by 1 mL of deionized water and carefully drained using a pipette, producing a stabilized suspension of approximately 30 mg/mL of IMQ. The concentration of IMQ was concurrently determined by UV-VIS spectroscopy, having used the values of absorbance at the wavelength of 242 nm (see Supplementary information for details). The nanocrystal suspension was subsequently diluted by deionized water to obtain 2 wt% IMQ, which was stored at 4 °C in a dark container.

#### 2.3.3 Preparation of samples containing polymers

In the case of nanoparticle formulations, **P1 – P3** polymers were dispersed in 2% concentration in the prepared nanoparticle formulation (NE, NC) until the polymers were dissolved.

For comparison purposes, a 2 wt% IMQ suspension was prepared by dispersing raw IMQ powder into a 2 wt% **P1–P3** solution in distilled water, followed by vortex mixing.

### 2.4 Characterisation of formulations containing polymers

#### 2.4.1 Particle size, polydispersity and zeta-potential

Particle size and zeta potential (ZP) were characterized by dynamic light scattering (DLS) using a Zetasizer Nano ZS instrument (Malvern Instruments, Worcestershire, UK) according to previous protocol (Petrová et al., 2023). For each measurement, 10 μL of the sample was diluted in 1 mL of filtered distilled water. The measurements were carried out at 25 °C with a scattering angle of 173°, and each analysis consisted of three runs with five scans per run. ZP was measured using disposable folded capillary cell. The approximation was performed with Henry's function and the Smoluchowski model. Three sets of measurement were taken, and the duration was automatically set, with the number of runs ranging from 10 to 100. The resulting data provided particle size (*Z*-average value), polydispersity index (PDI) and ZP values.

#### 2.4.2 Transmission electron microscopy

The NE samples (separately or in combination with 2% **P1**) were analysed by Jeol JEM-1010, Jeol Ltd., Tokyo, Japan (accelerating voltage: 80 kV). The NC samples (separately or in combination with 2% **P1**) were analysed using Jeol 2200 FS, Jeol Ltd., Tokyo, Japan (accelerating voltage: to 200 kV; vis. Point resolution 2.4 Å), equipped with TVIPS camera with EMenu sofware. Microscopy procedure was conducted following the protocol published in our previous study (Petrová et al., 2023).

The samples were prepared as follows: 10 μL of diluted suspension (1:5) of nanomaterial was deposited onto a carbon-coated copper mesh grid (Lacey Formvar/Carbon, 300 mesh, Cu, Ted Pella, CA, USA). After 15 min of free evaporation and adhesion of particles, the rest of water was removed. Before the TEM measurements the NE samples were exposed to a 1% solution of uranyl acetate for 5 min.

### 2.5 *Ex vivo* skin permeation study

#### 2.5.1 Permeation experiment setup

Fresh porcine skin was obtained from a local slaughterhouse. It originated from 6-month-old pigs of both sexes, with an approximate 1:1 sex ratio. The skin was isolated by blunt dissection from the dorsal side of the ears, cleaned in distilled water, and stored at −20 °C for a maximum period of three months.

The experiments were performed in unjacketed Franz diffusion cells (SES Analysesysteme, Bechenheim, Germany). The acceptor part (5 mL) was filled with PBS buffer (pH = 7.4) with gentamicin sulphate (50 mg/L) for microbial protection to mitigate potential bacterial decomposition of the skin during the experiment (Kováčik et al., 2020; Petrová et al., 2023; FDA, 2020). The skin (permeation area 1.0 cm^2^) was placed between the donor and acceptor parts. For equilibration, the cells were tempered at 32 °C ± 0.5 °C in a water bath for at least 1 h. Subsequently, the donor was filled with 300 μL of tested or control solution, which was present in the donor throughout the whole experiment (Dvořáková et al., 2022; Petrová et al., 2023).

#### 2.5.2 *V*isualization of polymer skin penetration by confocal microscopy

The **FP1 – FP3** polymers (5 wt% solution in distilled water) were applied on porcine skin in Franz diffusion cells. After 24 h, the skin was gently cleaned and cut in cross-section into 10 μm slices by cryostat (Leica CM1850, Deer Park, USA). The penetration depth of the polymers was observed by confocal microscopy (Olympus Fluoview FV1000, using UPLFLN 20× objective, Tokyo, Japan) according to a previous protocol (Kalvodová and Zbytovská, 2022). The samples were imaged with a laser (λ = 488 nm). The used laser strength was 2% and fluorescence detector gain (photomultiplier detector voltage 500 V) was not changed during the whole measurement (its value was 1).

##### 2.5.2.1 Image analysis

The profile of **FP1** – **FP3** content in the skin used for permeation experiments was determined *via* image analysis using open software Fuji/ImageJ (Schindelin et al., 2019). First, the background intensity was determined from images of non-treated skin as follows. All three colour channels (red, green, and blue) were separately converted to an 8-bit image, and the plot profile of colour intensity was obtained. Next, the average value of each colour channel was subsequently subtracted from images of skin treated with **FP1** – **FP3.** Finally, the green channel was chosen for the article as the most representative.

#### 2.5.3 Polymer effects on the skin tissue

##### 2.5.3.1 Polymers effects on the SC studied by Fourier-Transform Infrared Spectroscopy (FTIR)

To monitor effects of the polymers on the skin barrier, 300 μL of **P1-P3** water dispersion (2–5%) or distilled water (control) were applied on the skin surface in the Franz diffusion cells. After 24 h, the skin was gently washed with distilled water and dried by a cotton swab. The FTIR measurements were performed according to a published protocol (Čuříková-Kindlová et al., 2019; Čuříková-Kindlová et al., 2022). Each skin sample was positioned on a single-reflection MIRacle ZnSe crystal (PIKE technologies, Madison, WI, USA) with the SC oriented toward the crystal surface and the spectra were recorded using an IR spectrometer (Nicolet iZ10, Thermo Scientific, Waltham, MA, USA). All samples were measured within 1 h after removing the formulation. The spectra were generated at room temperature by co-addition of 64 scans collected at a resolution of 2 cm^−1^ and evaluated by OMNIC™ software (Thermo Scientific, Waltham, MA, USA). The spectra were normalized in the OriginPro software (OriginLab, Wellesley Hills, MA, USA) by its inbuilt “normalize” function to minimize the influence of variations of the total intensity of the individual measurements. The effects of tested formulations of the skin were evaluated by comparison of the position of characteristic peaks, which were determined in OriginPro software by its inbuilt “Find Peak” function.

##### 2.5.3.2 Reversibility of the skin barrier function measured by transepidermal water loss

Reversibility of the skin barrier function was measured according to previously reported protocols (Kováčik et al., 2020; Dvořáková et al., 2022). After the equilibration of the skin tissue in cells, the transepidermal water loss (TEWL) was measured by AquaFlux AF 200 (Biox Systems Ltd., London, Great Britain). Subsequently, 300 μL of **P1** - **P3** dispersed in water (2–25 wt%) or distilled water as a control were gently added into the donor part. After 24 h, the solutions were gently remo*v*ed, and TEWL values were monitored after 2, and 24 h.

#### 2.5.4 IMQ skin permeation experiment

IMQ permeation study was performed according to a previously described protocol (Petrová et al., 2023). Briefly, 300 μL of the donor sample (2% IMQ suspension, NE or NC with or without the specific polymer; Aldara®) were applied on the skin for 48 h. Afterward, the acceptor solution was taken from each cell and analysed by HPLC (see below for details). Then, the skin was removed, two times stripped and extracted in 3 mL of methanol:acetate buffer (20 mM, pH = 4) in ratio 7:3. The extracts were filtered through 0.22 μm filters.

##### 2.5.4.1 HPLC analysis

All the samples were analysed by Prominence LC-20 HPLC (Shimadzu, Tokyo, Japan) equipped with following: reverse-phase column Kinetex® (150 × 4.6 mm, 5 μm, RP C18, 100 Å) (Phenomenex, Torrance, USA); LC-20AD solvent delivery module with DGU-20A degasser; SIL-20AC autosampler; CTO-20AC column oven; SPD-M20A UV/VIS photodiode array detector; CBM-20A communication module. The mobile phase was acetonitrile:acetate buffer (pH = 4, 20 mM) in a 3:7 ratio (*v*/v). The flow was 1 mL/min, sample injection volume 20 μL, detection wavelength 242 nm, and retention time 4.5 min. A calibration curve was created from standards, and the exact IMQ concentration was calculated (see Supplementary information for details). LCsolution software (1.11 SP1) was used to evaluate the data. The HPLC method was validated in-house for linearity, accuracy and precision: the lower limit of detection (LLOD) was 0.67 μg/mL and the lower limit of quantification (LLOQ) was 2.04 μg/mL (see Supplementary information).

##### 2.5.4.2 Electrical impedance measurements

The formulations´ ability to impair the skin barrier function was evaluated by electrical impedance (EI) (Čuříková et al., 2017; Fasano and Hinderliter, 2004; Petrová et al., 2023; Vovesná et al., 2021). The EI of each skin was measured using a hand-held multimeter (LCR 4080, Voltcraft, Germany) set in parallel-equivalent mode with an alternating current frequency of 120 Hz. After the skin equilibration, the donor compartment was filled with PBS. One wire of the multimeter was placed in the donor, the other one in the acceptor phase, and EI was determined. Then, 300 μL of tested/control solutions were applied for 48 h. The next measurement was taken after the replacing of tested solutions with PBS buffers. The effect of formulations was e*v*aluated by determination of EI ratios obtained before and after 48-h exposure to the tested/control solutions.

### 2.6 Statistical analysis

Statistical analysis was performed using GraphPad Prism software (La Jolla, CA, USA). The Grubbs' test was used to identify the outlier values. One-way ANOVA analysis with Dunnetts' post-test was used. The differences were considered significant at *p* < 0.05. The data are presented as the mean values ± SEM.

## 3 Results and discussion

### 3.1 Synthesis and characterisation of polymers and copolymers

Three p(HPMA) homopolymers differing significantly in molecular weight and hydrodynamic size were synthesized (Table 1). Polymer **P1**, consisting of only about 35 monomer units, showed the lowest molecular weight and hydrodynamic radius. The molecular weight and size further increased significantly for polymers **P2**, and **P3**, comprising of about 130 or 500 monomer units, respectively. However, their dispersity was quite low due the utilization of the controlled radical RAFT polymerization.

**Table 1:** Physicochemical characteristics of the prepared p(HPMA) homopolymers.

Polymer	*M*_w_ (g·mol^−1^)^a^	*Ð*^a^	*R*_h_ (nm)^b^
**P1**	5300	1.1	2.4 ± 0.1
**P2**	20,300	1.1	3.7 ± 0.2
**P3**	80,000	1.2	7.8 ± 0.2

a The weight-average molecular weight (*M*_w_) and the dispersity (*Đ*) of the polymers were determined by GPC using MALS and RI detectors and TSKgel SuperSW3000 column in methanol and 0.3 M sodium acetate buffer at pH 6.5 (8/2, *v*/*v*).

b The hydrodynamic radius (R_h_) was measured in PBS (pH 7.4) at 2 mg/mL using a Nano-ZS instrument (Malvern) with a laser at λ = 632.8 nm. The intensity of scattered light was detected at *θ* = 173°.

Fluorescently labelled polymer analogues **FP1** – **FP3** were prepared from the polymer precursors **P1-TT** – **P3-TT,** synthesized by copolymerisation of HPMA and a monomer bearing TT reactive groups (MA-AP-TT). The P1-TT – P3-TT had almost the same physicochemical characteristics as homopolymers **P1** – **P3** (Table 2), thus it was possible to correlate the data obtained from both series of samples to each other without any difficulty. The content of TT groups (2 mol%) was sufficient for the attachment of the fluorescent dye DY-490 (about 2 wt%). Although the precise molecular weight of **FP1** – **FP3** copolymers could not be determined using MALS detection due to the fluorescence of the attached dye, the RI chromatograms corresponded with those of the polymers without the dye. Thus, we can conclude that the molecular weight and dispersity did not change during the reaction.

**Table 2:** Physicochemical characteristics of the prepared fluorescently labelled polymers.

Sample	Prepared from	*M*_w_ (g·mol^−1^)^a^	*Ð*^a^	*R*_h_ (nm)^b^	DY-490 content (wt%)^c^
**FP1**	**P1-TT**	5700	1.2	1.9 ± 0.1	2.1
**FP2**	**P2-TT**	23,200	1.1	3.9 ± 0.2	1.7
**FP3**	**P3-TT**	86,100	1.2	8.2 ± 0.4	2.0

a The weight-average molecular weight (*M*_w_) and the dispersity (*Đ*) of the polymer precursors were determined by GPC using MALS and RI detectors and TSKgel SuperSW3000 column in methanol and 0.3 M sodium acetate buffer at pH 6.5 (8/2, *v*/*v*).

b The hydrodynamic radius (*R*_h_) was measured in PBS (pH 7.4) at 2 mg/mL using a Nano-ZS instrument (Malvern) with a laser at *λ* = 632.8 nm. The intensity of scattered light was detected at *θ* = 173°.

c The content of the dye was determined by UV–Vis spectrophotometry.

### 3.2 HPMA polymers penetrate the skin depending on their molecular size

**Fig. 2:**
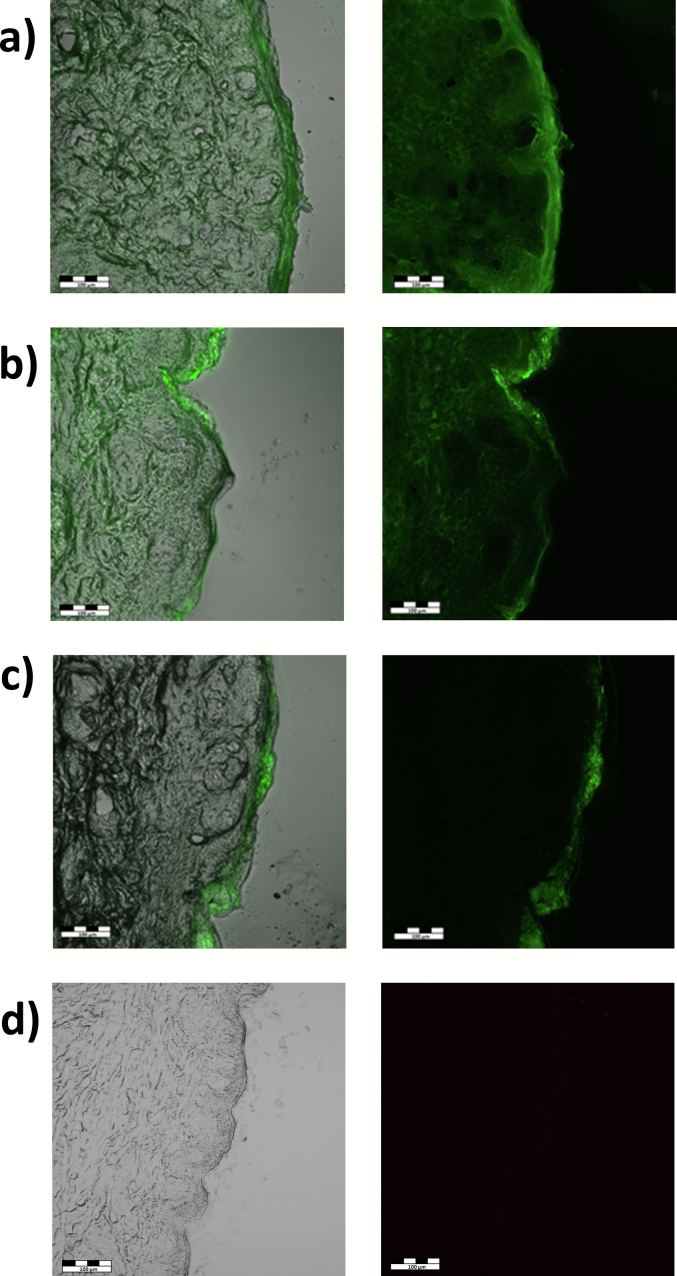
Cross-sectional optical and fluorescence microscopy images of porcine skin after 24 h of incubation in Franz cells by different donor solutions: FP1 (a), FP2 (b), FP3 (c), all as 5 wt% solution in distilled water, and control – distilled water (d). The scale bar represents 100 μm.

Fluorescently labelled polymers **FP1** – **FP3** were used to visualise their penetration into the skin after a 24-h incubation (Fig. 2). At the wavelength of λ = 488 nm, there was no skin autofluorescence (see Fig. 2d), which implied that all visible signals came from the fluorescently labelled polymer.

**Fig. 3:**
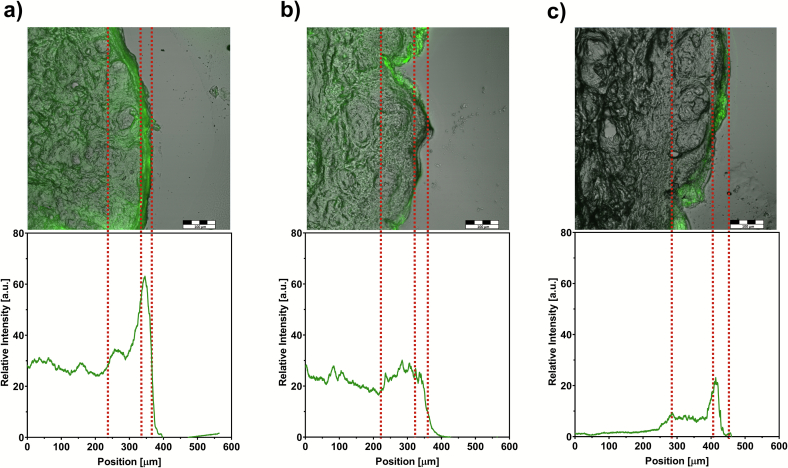
The semi-quantitative analysis of polymer distribution in porcine skin presented by relative intensity of fluorescent signal obtained by the confocal microscope, the red dot lines mark the stratum corneum and epidermis: FP1 (a), FP2 (b), and FP3 (c). Scale bar = 100 μm. (For interpretation of the references to colour in this figure legend, the reader is referred to the web version of this article.)

Fig. 2, Fig. 3 clearly demonstrate that penetration of p(HPMA) polymers corresponds to their molecular weight which closely correlates with the hydrodynamic size. This suggestion was confirmed by the semi-quantitative analysis of the confocal images (Fig. 3). The high molecular-weight polymer **FP3** (86100 g/mol) exhibited minimal penetration. It reached fluorescence intensity of around 23 a.u. on the skin surface. In the deeper layers of the epidermis, the signal intensity decreased, reaching baseline levels. Taking into account the thickness of the SC on porcine ear skin (≈20 μm) (Meyer and Zschemisch, 2002), it can be concluded that this polymer penetrated only partially the SC but not deeper. Moreover, the signal within the SC was distributed rather heterogeneously. In contrast, the medium-sized polymer **FP2** (23200 g/mol) showed significantly greater penetration with a homogenously distributed signal throughout the skin section. At the skin surface, the intensity value reached around 26 a.u. and the signal was uniform within the whole skin tissue. A similar pattern was also observed for the smallest **FP1** (5700 g/mol)**,** but with substantially higher intensity. Within the SC, the fluorescent intensity reached approximately 62 a.u. and was very uniformly distributed. In the deeper skin layers, the intensity decreased to around 30 a.u. throughout the entire sample. Among all tested polymers, **FP1** demonstrated the greatest ability to permeate the SC. This enhanced permeation is likely due to the small hydrodynamic size of **FP1** and possibly better solubility in skin tissue, which may facilitate its transport across the skin barrier. Similar behaviour has already been observed for other hydrophilic polymers. For example, low molecular weight hyaluronic acid (5000 g/mol) demonstrated substantially enhanced penetration across the skin barrier relative to higher-molecular-weight forms (Essendoubi et al., 2016; Witting et al., 2015).

In dermal drug delivery, the ability to modulate polymer penetration depth by its molecular size represents a key parameter in the formulation design. Thus, depending on the targeted skin layer, it can be possible to tailor the optimal polymer size to achieve the desirable depth of the drug distribution (Paradee et al., 2021; Witting et al., 2015).

### 3.3 P(HPMA) increase hydration of the SC

Given the proven penetration of p(HPMA) into deeper layers of the skin, we focused on the question of whether the polymer interacts with the skin barrier located in the SC and whether this can be detected at the molecular level. Measurement of skin FTIR spectra was used to monitor the effect of HPMA on the both parts of SC, namely the lipid matrix and keratin-rich corneocytes (Roger et al., 2019). The polymer solutions in water (2–5%) were applied to the skin for 24 h and after their removal, the FTIR spectrum was recorded from each skin sample. The effect of p(HPMA) was interpreted according to the evaluation published by Olsztyńska-Janus et al. (Olsztyńska-Janus et al., 2018). In the IR spectra of skin surface, both lipids and proteins can be monitored in separated vibration modes.

**Fig. 4:**
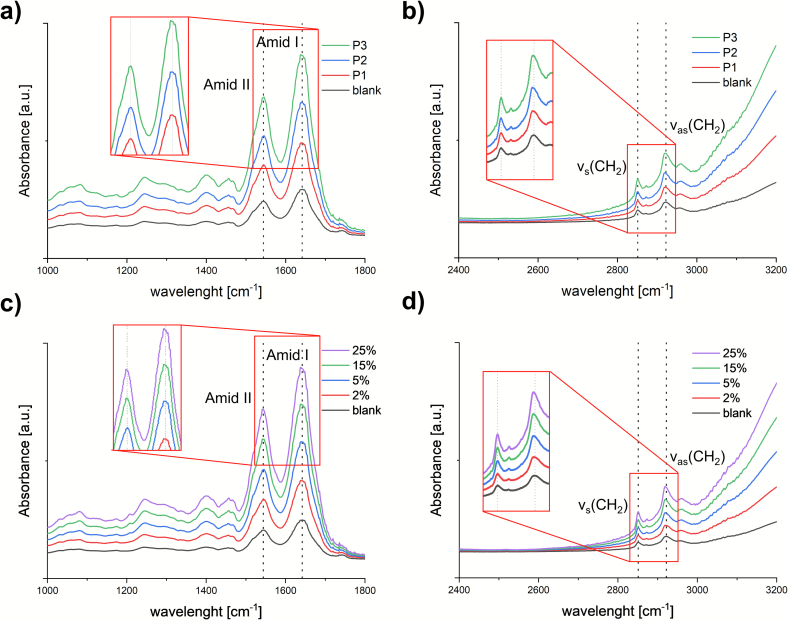
ATR-FTIR spectra of skin treated with P1-P3 in 2% water dispersion in the spectral range 1000–1800 cm^−1^ with enlarged amid I and II peaks (a) and 2400–3200 cm^−1^(b); skin treated with different concentrations of P1 in water in the spectral range 1000–1800 cm^−1^ with enlarged amid I and II peaks (c) and in the spectral range 2400–3200 cm^−1^ (d). The dashed lines show the positions of amide I/II and ν_a/as_(CH_2_); control: blank skin treated with pure water.

Symmetric (∼2850 cm^−1^) and asymmetric (∼2920 cm^−1^) stretching CH_2_ vibrations (ν_s/as_CH_2_) were the main modes of interest to uncover the arrangement of the hydrocarbon chains of SC lipids, especially ceramides. A shift to higher wavenumbers suggests higher mobility of the chains and thus increased fluidity of the membranes (Čuříková-Kindlová et al., 2019)*.* According to the values in Table 3 and Fig. 4ab, there was no significant difference between the skin samples treated with the 2 wt% **P1-P3** water dispersions and the blank sample (distilled water) which indicated that p(HPMA) did not affect the arrangement of the SC lipids.

**Table 3:** Positions of Amide *I*, II, and CH_2_ stretching vibrations of the skin surface after application of different p(HPMA) solutions (P1-P3, 2% in water). Control is blank skin treated by distilled water; *n* = 3.

	Amide *I*	Amide II	ν_s_CH_2_	ν_as_CH_2_
Blank	1641.8 ± 0.9	1544.4 ± 0.9	2850.8 ± 0.4	2922.1 ± 0.9
**P1**	1639.4 ± 1.7	1544.4 ± 0.9	2850.4 ± 0.0	2918.1 ± 0.7
**P2**	1641.1 ± 0.7	1544.3 ± 0.9	2850.1 ± 0.3	2917.9 ± 0.6
**P3**	1637.8 ± 1.4	1541.1 ± 1.2	2850.4 ± 0.0	2922.7 ± 0.0

Effects on the corneocytes could be described by the shift in the position of amide bands, specifically amide I (C=O stretch; ∼1640 cm^−1^) and amide II (N—H in-plane bend, C—N stretch; ∼1540 cm^−1^). These signals are known as very sensitive to modifications in protein structure. A shift to lower wavenumbers corresponds to changes in hydrogen bonds of the proteins (Olsztyńska-Janus et al., 2018). There was no significant shift between the blank samples and skin treated with the **P1** and **P2** type. In the case of **P3**, a slight shift to 1637.8 cm^−1^ (see Table 3) could suggest changes in keratin secondary structure indicating a loose of accumulation of SC proteins and higher permeability of SC (Covi-Schwarz et al., 2017).

A similar experiment was also performed for different concentrations of the **P1** polymer. The results are summarized in Table 4 and Fig. 4 c, d. Each vibration position was compared to the corresponding position of the blank sample (skin treated with distilled water). Once again, it was evident that there was no interaction between **P1** and the SC lipids, as the CH_2_ vibration did not exhibit a shift in position. However, significant changes were recognized in the region of amide vibrations. These shifts were observable when the solutions contained **P1** in 15% or more. In these cases, a shift toward lower wavenumbers was detected, reflecting changes in keratin secondary structure which potentially facilitated the higher permeability of SC (Covi-Schwarz et al., 2017; Olsztyńska-Janus et al., 2018).

**Table 4:** The positions of Amide *I*, Amide II, and CH_2_ stretching vibrations of the skin surface after treatment with P1 in water dispersion at various concentration. Control is blank skin treated by distilled water. n = 3.

	Amide I	Amide II	ν_s_CH_2_	ν_as_CH_2_
Blank	1641.8 ± 0.9	1544.4 ± 0.9	2850.8 ± 0.4	2922.1 ± 0.9
5	1640.3 ± 1.1	1543.8 ± 0.9	2849.9 ± 0.3	2917.6 ± 0.3
15	1636.4 ± 1.5	1540.9 ± 0.3	2850.1 ± 0.3	2917.9 ± 0.0
25	1636.4 ± 0.0	1539.3 ± 0.0	2849.4 ± 0.0	2917.9 ± 0.0

**Fig. 5:**
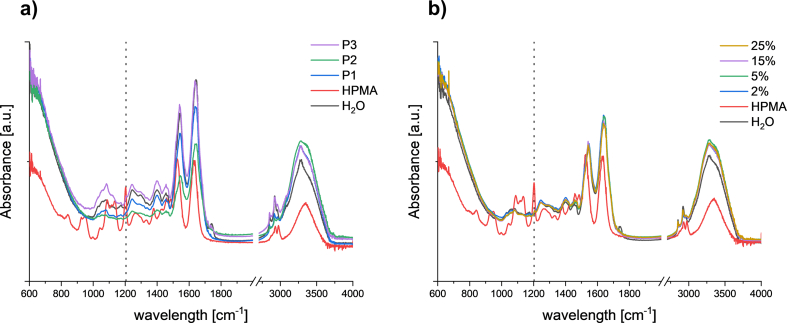
ATR-FTIR spectra of the skin treated with P1-P3 in 2% water dispersion (a), and with P1 water dispersion of different concentrations (b). The dashed line shows the mode position characteristic for p(HPMA); controls: distilled water, pure P1 powder (HPMA).

In addition, to evaluate the effects of p(HPMA) on corneocytes and lipid matrix, FTIR can also be used to assess the degree of SC hydration using the water characteristic bands (OH valence vibration at 3100–3500 cm^−1^ (Olsztyńska-Janus et al., 2018)). When examining the individual spectra for the skin treated by **P1-P3** aqueous dispersion (2%) (Fig. 5a) and different concentrations of **P1** (Fig. 5b), there was a noticeable difference between the band intensity for the control (distilled water) and for the samples containing p(HPMA). In the presence of p(HPMA) the band intensity increased. This increase was not caused by the presence of p(HPMA) itself. Comparing the spectra for pure p(HPMA) with the others, it was evident that the position for the OH valence vibration shifted to lower wavenumbers, and, moreover, the p(HPMA) characteristic band at 1200 cm^−1^ was not visible. Taken together with the shifts observed in the amide region, these findings indicate that the applied p(HPMA) increased the hydration of the SC, particularly affecting keratin within the corneocytes. This phenomenon was also observed for another hydrophilic polymer, hyaluronic acid, as described by Witting et al. (Witting et al., 2015).

### 3.4 The effects on the skin barrier are reversible

As described above, FTIR spectroscopy provided molecular-level information on the interaction between p(HPMA) and SC. The presence of p(HPMA) was found to modulate skin hydration and influence keratin organization within the SC. In the next phase of the research, the potential effects of these manifestations on the functionality of the skin barrier, for example through increased permeability, were examined, as well as the reversibility of these changes. For this purpose, the transepidermal water loss (TEWL) was employed, serving as a functional indicator of skin barrier integrity. This method is used *in vivo* as well as *ex vivo* to evaluate the amount of water passively evaporating through the skin, which reflects the skin barrier integrity (Alexander et al., 2018; Guth et al., 2015; Hattingh, 1972).

**Fig. 6:**
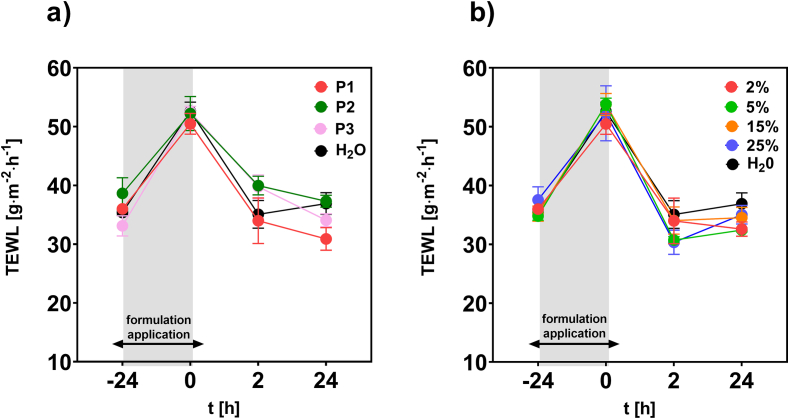
The TEWL values of the porcine skin *ex vivo* before and after application of P1 – P3 at 2 wt% in water (a) and for different concentrations (2–25 wt%) of P1 (b); *n* = 3.

After the skin surface was incubated for 24 h by **P1-P3** polymers at 2 wt% aqueous solutions, its TEWL values increased similarly as in the case of control (distilled water) (Fig. 6a). However, the values decreased back to the initial ones within 2 h after the removal of polymer solutions and did not differ for that obtained for the control. These results suggested that p(HPMA) did not affect the skin barrier properties more than distilled water.

Moreover, even the concentrated solutions (up to 25 wt%) of **P1** did not show any harmful effect on the skin barrier function (Fig. 6b). The full recovery was observed again within 2 h after the formulation removal. The absence of harmful effects on the skin barrier is fully consistent with the high biocompatibility and non-fouling characteristics of p(HPMA) reported elsewhere. p(HPMA) polymers also exhibited no significant interactions with series of the most abundant component of the human plasma (Klepac et al., 2018) and they showed excellent properties even in the compassionate use in humans (Dozono et al., 2016).

### 3.5 Nanoparticulate formulations combined with p(HPMA)

The previous results showed that p(HPMA) polymers penetrated the skin with respect to their hydrodynamic size and that they show hydrating effects on the skin barrier. This finding led us to consider whether this property might also enhance the penetration of an active substance into the skin. To explore this, a combined formulation using p(HPMA) and nanoparticles loaded with the active ingredient IMQ was prepared. For this purpose, we applied previously developed nanoparticulate formulations, namely nanoemulsion (NE) and nanocrystals (NC), which had already demonstrated a strong ability to enhance the skin penetration of IMQ (Petrová et al., 2023).

**Fig. 7:**
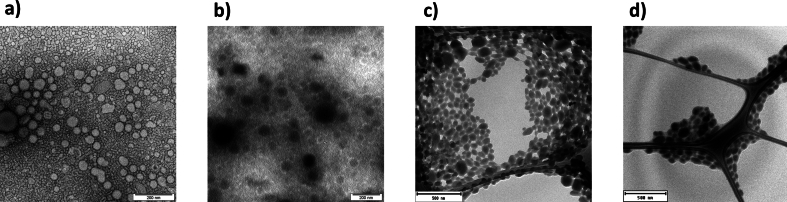
TEM images of the combined samples: nanoemulsion (a), nanoemulsion in combination with P1 (2 wt%) (b), nanocrystals (c) and nanocrystals in combination with P1 (2 wt%) (d). Scale bar = 200 nm (a, b); 500 nm (c, d). The elongated lines visible in (c) and (d) originate from the underlying microscopic grid.

To assess the potential impact of p(HPMA) on the physicochemical properties of nanoparticulate formulations, nanoparticles either containing or lacking **P1** were characterized with respect to their hydrodynamic size, dispersity, surface charge, and morphology (Table 5, Fig. 7).

**Table 5:** Comparative summary of the key physicochemical characteristics of nanoemulsion (NE) and nanocrystals (NC) formulations, with and without 2% P1.

Parameter	NE	NE+2%**P1**	NC	NC + 2% **P1**
size [nm]	118.5 ± 0.4	118.6 ± 0.6	122.0 ± 3.8	124.5 ± 1.0
PDI [−]	0.12 ± 0.01	0.12 ± 0.01	0.18 ± 0.01	0.18 ± 0.03
zeta potential [mV]	−22.8 ± 0.3	−21.7 ± 0.4	−16.1 ± 0.6	−13.0 ± 0.9

For the NE formulation, the incorporation of **P1** did not lead to measurable changes in particle size, dispersity, or zeta potential. Both NE variants displayed similarly low PDI values and comparable negative surface charge, which remained stable over a two-week observation period. TEM micrographs (Fig. 7a, b) confirmed that NE particles maintained a spherical morphology with a consistent size distribution, independent of the presence of **P1**. These findings indicate that **P1** did not affect the structural characteristics of NE.

A comparable trend was observed for NC. The mean particle size and PDI values were nearly identical for both NC variants. Although a very slight decrease in the absolute value of zeta potential was detected upon **P1** incorporation, the magnitude of this shift was minimal and did not affect overall physical characteristics. Moreover, the values remained stable during the two-week stability assessment. TEM analysis (Fig. 7 c,d) revealed oblong particles with a similar size range in both formulations correlating with the DLS results.

Collectively, these results demonstrate that **P1** incorporation did not significantly affect the size distribution, surface charge, or morphology of either NE or NC formulations.

### 3.6 P(HPMA) polymers enhance IMQ penetration into the skin tissue

The effectiveness of combined nanoformulations with p(HPMA) was assessed through *ex vivo* permeation experiments using porcine skin. These experiments were conducted under the same conditions as previous permeation studies (Kalvodová and Zbytovská, 2022; Petrová et al., 2023; Petrová et al., 2025) and involved formulations containing 2 wt% IMQ (maximum IMQ loading into the NE (Petrová et al., 2023) and 2 wt% p(HPMA), namely nanocrystals (P1/2/3-NC) or nanoemulsions (P1/2/3-NE). For comparative purposes, we included a simple IMQ suspension in distilled water (P1/2/3-IMQ-H₂O) as controls. Our main goal was to achieve the highest possible concentration of IMQ in skin tissue with minimal transdermal penetration which is generally undesirable due to systemic side effects (Hanna et al., 2016). Therefore, we also used the commercially available Aldara cream (containing 5% IMQ) as a marker for comparison of the results.

**Fig. 8:**
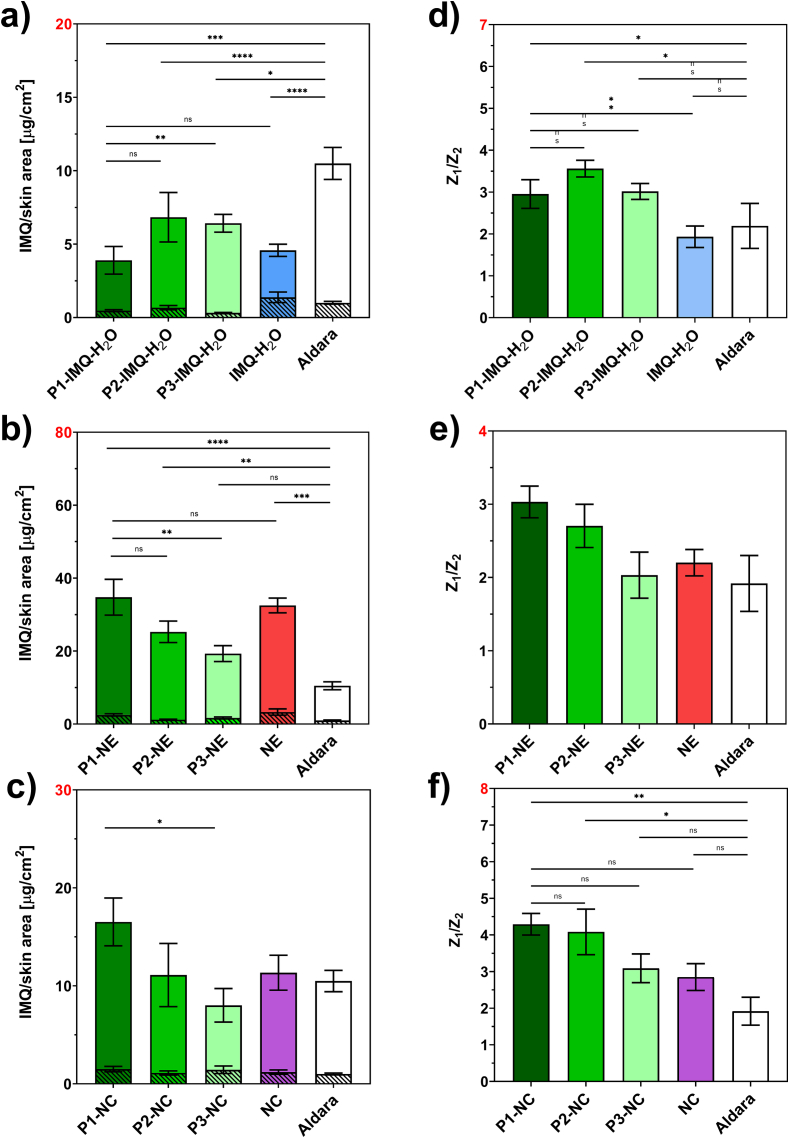
Effects of different p(HPMA) on the skin uptake and permeation of IMQ and skin barrier integrity expressed by the EI ratio. a-c show the accumulation of IMQ in the acceptor phase (patterned columns) and in the skin tissue (solid columns). d-e show the impedance ratio describing the level of skin barrier integrity. The green colour represents the combined systems with the P1-P3 polymers, the blue colour represents simple IMQ suspension in water, the red one pure NE, and purple one pure NC. The number of asterisks shows the level of significance for comparison of respective formulations. (p < *0.05, **0.01, ***0.001 and ****0.0001); the values in panel (e) do not exhibit statistically significant differences against control; *n* ≥ 6. (For interpretation of the references to colour in this figure legend, the reader is referred to the web version of this article.)

The results for IMQ entrapment within the skin, IMQ permeation amount into the acceptor phase, and the corresponding impedance ratio, reflecting the extent of skin barrier disruption, are presented in Fig. 8. Table 6 summarizes the quantitative data for IMQ entrapment and permeation. These findings are further discussed in the following subsections.

**Table 6:** Dermal entrapment and permeated amount of IMQ after a 48-h permeation process from suspension without (IMQ-H_2_O) and with p(HPMA) of different size (P1-P3-IMQ-H_2_O), nanoemulsion without (NE) and with p(HPMA) (P1-P3-NE), nanocrystals without (NC) and with p(HPMA) (P1-P3-NC), and the marketed cream Aldara.

Formulation	Dermal entrapment [μg/cm^2^]	Permeated amount [μg/cm^2^]
P1-IMQ-H_2_O	3.43 ± 0.94	0.47 ± 0.07
P2-IMQ-H_2_O	6.16 ± 1.69	0.67 ± 0.15
P3-IMQ-H_2_O	6.10 ± 0.61	0.32 ± 0.03
IMQ-H_2_O	3.20 ± 0.42	1.38 ± 0.36
P1-NE	32.25 ± 4.91	2.52 ± 0.31
P2-NE	24.08 ± 2.95	1.20 ± 0.12
P3-NE	17.64 ± 2.17	1.68 ± 0.27
NE	29.23 ± 2.03	3.29 ± 0.87
P1-NC	15.03 ± 2.44	1.50 ± 0.28
P2-NC	9.99 ± 3.22	1.11 ± 0.21
P3-NC	6.58 ± 1.71	1.43 ± 0.38
NC	10.15 ± 1.79	1.19 ± 0.22
Aldara	9.49 ± 1.09	1.00 ± 0.10
Data are presented as means ± SEM; *n* ≥ 6		

#### 3.6.1 Suspension

First, a simple IMQ suspension was evaluated in the presence of 2% p(HPMA), using the IMQ suspension prepared in distilled water as the control. The amounts of IMQ retained in the skin and permeated into the acceptor phase are shown in Fig. 8a. Transdermal permeation was low for both, the polymer-containing formulations and the control, reaching 1.38 ± 0.36 μg/cm^2^ for the suspension in pure water. The addition of p(HPMA) further reduced permeation to less than half of this value.

In contrast, more pronounced differences were observed in dermal uptake. The control IMQ suspension exhibited a dermal entrapment of 3.20 ± 0.42 μg/cm^2^. The presence of p(HPMA) consistently increased IMQ retention in the skin regardless of polymer type, with the effect being particularly evident for higher–molecular-weight polymers (**P2** and **P3**), where dermal entrapment nearly doubled relative to the control.

Although p(HPMA) enhanced the dermal uptake of IMQ, the overall amount of IMQ released from the polymer-containing suspensions remained lower than that observed for the Aldara cream. This discrepancy is likely due to both formulations relying on a conventional suspension system and the substantially higher IMQ concentration in the commercial cream (5%) compared with the 2% concentration used in our samples.

The potential disruption of the skin barrier was evaluated by EI (Fig. 8d). Higher impedance ratios for the samples containing p(HPMA) compared to the control suggest a reduction in skin barrier resistance to the flow of small, charged molecules (*e.g.*, ions), indicating a temporary weakening of the skin barrier. This weakening correlates with the uncovered SC hydration effects of p(HPMA) and may explain the enhancing effects of p(HPMA) for IMQ. It should be noted that the reversibility of the effects was already confirmed by TEWL.

#### 3.6.2 Nanoemulsions

All NE formulations showed markedly enhanced dermal retention relative to both the suspension and the Aldara benchmark (Fig. 8b). The pure NE formulation achieved a retention level of 29.23 ± 2.03 μg/cm^2^, representing a threefold increase compared with Aldara (9.49 ± 1.09 μg/cm^2^). In contrast to their pronounced acceleration effect observed with the conventional suspension, the polymers did not substantially enhance dermal deposition when incorporated into the NE system. Only polymer **P1** produced a slight, statistically non-significant increase (32.25 ± 4.91 μg/cm^2^), while **P2** and **P3** exhibited even inhibitory effects. A more favourable influence of the polymers became apparent in the context of transdermal permeation. Compared with the pure NE, all polymer-containing formulations reduced permeation, although these reductions did not reach statistical significance.

The impedance ratio, used as an indicator of skin barrier permeability to small charged molecules, was similar for the pure NE formulation and the Aldara cream (Fig. 8e). Notably, formulations with polymers **P1** and **P2** showed increases of approximately 30%, which correlates well with the FTIR observation that they hydrate the skin tissue.

#### 3.6.3 Nanocrystals

Compared with the suspension, NC exhibited a stronger ability to deliver IMQ into the skin tissue, although the enhancement was less pronounced than that observed for NE. The pure NC formulation achieved 10.15 ± 1.79 μg/cm^2^, a value comparable to that of Aldara; however, it is important to note that NC contained only 2% IMQ, whereas Aldara contained 5%. The incorporation of p(HPMA) further affected IMQ dermal penetration from the NC formulations (Fig. 8c). While **P2** and **P3** showed rather negative effects on dermal IMQ retention, **P1** acted as an effective enhancer, increasing entrapment by approximately 50% to 15.03 ± 2.44 μg/cm^2^. Notably, the addition of **P1–P3** did not lead to any statistically significant increase in unwanted transdermal permeation, which represents an additional advantage.

As indicated by the EI ratio, skin-barrier permeability to small charged molecules was particularly affected in the P1-NC and P2-NC formulations (Fig. 8f). This effect is consistent with the findings obtained for the other formulations and correlates well with both the dermal entrapment data and the FTIR results, which confirmed enhanced hydration of the skin barrier mediated by the polymers.

#### 3.6.4 Summary of the ex vivo permeation experiments

In recent years, increasing attention has been directed toward the use of hydrophilic polymers as enhancers of dermal and, in some cases, transdermal drug penetration (Lee et al., 2025). Several polysaccharides, such as hyaluronic acid and chitosan, have been shown not only to exhibit a remarkable ability to traverse the SC, but also to enhance the retention of co-administered drugs within skin tissue (Witting et al., 2015; Lee et al., 2024). These effects appear to be even more pronounced when the polymers are incorporated into nanoparticulate delivery systems. Together, these findings prompted us to explore a hydrophilic polymer outside the commonly studied polysaccharide class, specifically p(HPMA).

In the first part of our study, we demonstrated that the p(HPMA) is likewise capable of permeating the skin barrier in a molecular-weight-dependent manner while simultaneously increasing skin hydration. These properties suggested that p(HPMA) could be a promising candidate for enhancing the dermal penetration of active substances. To evaluate this potential, we selected the model compound IMQ and examined its delivery using both a conventional suspension and a nanoparticle-based formulation.

In summary, permeation experiments demonstrated that p(HPMA) is capable of enhancing the dermal penetration of IMQ across the skin barrier, both in conventional suspensions and in nanoparticle-based formulations. This effect was particularly pronounced in terms of dermal drug accumulation, while transdermal penetration was less affected, which is a favourable outcome in the context of minimizing potential systemic side effects of IMQ. The previously reported enhancement effect of IMQ in nanoformulations compared with conventional suspensions (Petrová et al., 2023) was also clearly observed. Notably, p(HPMA) exhibited a penetration-enhancing effect across all formulation types, including the suspension, NE, and NC. This effect appeared to be dependent on the polymer's molecular weight, with the smallest polymer, **P1** (5 kg/mol, hydration radius about 2 nm), proving most effective.

Compared with the NE, the enhancing effect was particularly pronounced in the NC system, where **P1** increased IMQ penetration into the skin by approximately 50% relative to the control. The penetration-enhancing ability of p(HPMA) correlated well with measurements of EI, where higher ratios indicated a temporary weakening of the skin barrier to small charged molecules, likely attributable to increased hydration of the skin tissue, as supported by FTIR analysis.

Taken together, these findings highlight the considerable potential of p(HPMA) as a functional excipient in topical formulations and cosmetic applications. Given its ability to improve dermal drug retention without significantly increasing systemic absorption, p(HPMA) emerges as a promising tool for the design of safer and more effective skin-targeted therapies.

## 4 Conclusion

Polymers based on *N*-(2-hydroxypropyl)methacrylamide, p(HPMA), are hydrophilic and biocompatible materials with broad applicability in biomedical fields. In this study, we demonstrate their potential in topical drug delivery. Using confocal microscopy, we confirmed that these polymers can penetrate the skin barrier and accumulate within the epidermis and dermis. This permeation capacity is highly dependent on their hydrodynamic size. Particularly, polymer **P3** (80 kg/mol; *R*_*h*_ ∼ 8.2 nm) showed minimal permeation, whereas its smaller analogue **P1** (5 kg/mol; *R*_*h*_ ∼ 1.9 nm) demonstrated significant transdermal penetration.

FTIR enabled us to investigate polymer–skin barrier interactions at a molecular level. While p(HPMA) did not disrupt the ordered lipid arrangement within the intercellular spaces of the SC, they markedly increased overall hydration of the SC. This hydration likely affected the secondary structure of keratin in SC corneocytes. Notably, this impact on the skin barrier was shown to be reversible, as confirmed by TEWL.

These beneficial effects on the skin barrier inspired the next phase of our study, in which p(HPMA) were employed as penetration enhancers for the topically active compound IMQ. All polymers exhibited enhancement effects, resulting in increased accumulation of IMQ in native skin tissue. The polymer with the lowest molecular weight proved to be the most effective in this regard. This enhancement was observed across both a traditional formulation (suspension) and nanoparticle-based systems (NE and NC). Importantly, these formulations achieved higher concentrations of IMQ in targeting skin tissue than a commercially available product, while using less than half the amount of IMQ.

Altogether, this study demonstrates that HPMA polymers are effective dermal absorption enhancers. Their tunable properties *via* RAFT polymerization also highlight their potential as versatile drug carriers, capable of selectively targeting specific layers of the skin and improving therapeutic outcomes.

## Declaration of generative AI in scientific writing

During the preparation of this manuscript the authors used Copilot for Microsoft 365 in order to improve the readability and language of the text. After using this tool, the authors reviewed and edited the content as needed and take full responsibility for the content of the published article.

## CRediT authorship contribution statement

**Eliška Kurfiřtová:** Writing – review & editing, Writing – original draft, Investigation, Formal analysis, Data curation. **Stanislav Chvíla:** Writing – review & editing, Validation, Methodology. **Nikola Strnádková:** Data curation, Investigation, Writing – review & editing. **Vendula Janoušková:** Data curation, Investigation, Writing – review & editing. **Petr Chytil:** Writing – review & editing, Validation, Methodology. **Tomáš Etrych:** Writing – review & editing, Funding acquisition, Conceptualization. **Jarmila Zbytovská:** Writing – review & editing, Validation, Supervision, Funding acquisition, Conceptualization.

## Funding sources

The work was supported by the project New Technologies for Translational Research in Pharmaceutical Sciences/NETPHARM, ID CZ.02.01.01/00/22_008/0004607, co-funded by the 10.13039/501100000780European Union. The work was supported by the project National Institute for Cancer Research (Programme EXCELES, ID Project No. LX22NPO5102) – Funded by the European Union – Next Generation EU and by the Czech Science Foundation (project 25-17203S).

## Declaration of competing interest

The authors declare the following financial interests/personal relationships which may be considered as potential competing interests:

Jarmila Zbytovska reports financial support was provided by Czech Science Foundation. Jarmila Zbytovska reports financial support was provided by European Union. Tomas Etrych reports financial support was provided by European Union. If there are other authors, they declare that they have no known competing financial interests or personal relationships that could have appeared to influence the work reported in this paper.

## Data Availability

The dataset is available in the Zenodo repository.

## Appendix A Supplementary data

Supplementary material Image 1
