# Supplementary material for: HPMA polymers as functional excipients in dermal nanoformulations of imiquimod
Source: Int J Pharm X. 2026 Jan 8;11:100486. doi: 10.1016/j.ijpx.2026.100486 (PMC12830183; doi:10.1016/j.ijpx.2026.100486)
Supplement: Supplementary file 1 — Supplementary material [file mmc1.docx]

# **Supplementary information**

# HPMA polymers as functional excipients in dermal nanoformulations of imiquimod

# Eliška Kurfiřtová^1^, Stanislav Chvíla^2^, Nikola Strnádková^1^, Vendula Janoušková^1^, Petr Chytil^3^*, Tomáš Etrych^3^, Jarmila Zbytovská^1^*

# University of Chemistry and Technology Prague, Faculty of Chemical Technology, Department of Organic Technology, Technická 5, 166 28 Prague, Czech Republic

# University of Chemistry and Technology Prague, Faculty of Chemical Engineering, Department of Chemical Engineering, Technická 5, 166 28 Prague, Czech Republic

# Institute of Macromolecular Chemistry, Czech Academy of Sciences, Heyrovského nám. 2, Prague 6, 162 06, Czech Republic

**Determination of IMQ concentration in nanocrystal samples by UV-VIS spectrometry**

In order to work up and dilute the nanocrystal suspensions of IMQ after their preparation, UV spectrometry was utilized according to Petrová et al, 2023.

Three precisely weighed charges of IMQ between 5 and 10 mg were each dispersed in a 100-mL volumetric flask in 50 mL of dry methanol (Fluka) and sonicated until fully dissolved for approximately 2 minutes. Then the flasks were left still at lab temperature, filled up to the 100-mL mark, sealed and shaken vigorously for 60 seconds. Then the absorbance of IMQ was measured in a quartz cuvette (Hellma QS-104 Macro, path length 10 mm, fill volume 2 mL) using a SPECORD 205 BU UV-VIS spectrometer.

An absorption peak at 243 nm was identified, and used for the purpose of calibration, yielding an expression of y = 62.71*x + 0.002, where y is the absorbance in arbitrary units and x is the concentration of imiquimod in mg/mL.

The same procedure was repeated in UV-grade ethanol (Lachner), giving an absorption peak of 242 nm, and slope and intercept values less than 3% different to those given above.

To measure the concentration of IMQ in the suspension, 50 μL of the sample was diluted in 5 mL of dry methanol in a glass vial, sealed and shaken vigorously until there was no turbidity visible (ca. 30 seconds), and then for 30 more seconds. Then, 50 μL of the first diluate was diluted in 5 mL of dry methanol and measured under conditions listed above. This procedure was repeated at least once more, the average mass concentration was calculated, and the desired volume of suspension was diluted to a working concentration listed in the article.

**HPLC method for imiquimod determination in *ex vivo* study**

All the samples were analysed by Prominence LC-20 HPLC (Shimadzu, Tokyo, Japan) equipped with following: reverse-phase column Kinetex® (150 × 4.6 mm, 5 µm, RP C18, 100 Å) (Phenomenex, Torrance, USA); LC-20AD solvent delivery module with DGU-20A degasser; SIL-20AC autosampler; CTO-20AC column oven; SPD-M20A UV/VIS photodiode array detector; CBM-20A communication module. The procedure was used identical as already published by our group (Petrová et al., 2023).

The mobile phase was acetonitrile/acetate buffer (pH = 4, 20 mM) in a 3:7 ratio (v/v). The flow was 1 mL/min, sample injection volume 20 μL, detection wavelength 242 nm, and retention time 4.5 min. A calibration curve (y=153593x-291313; R^2^ = 0.999) was created from 8 standards (200 μg/ml → 2 μg/ml), and the exact IMQ concentration was calculated. LCsolution software (1.11 SP1) was used to evaluate the data. The method was validated for linearity, accuracy and precision in house. The lower limit of detection (LLOD) was 0.67 µg/ml and the lower limit of quantification (LLOQ) was 2.04 µg/ml.

**References**

Petrová, E., Chvíla, S., Balouch, M., Štěpánek, F., Zbytovská, J., 2023. Nanoformulations for dermal delivery of Imiquimod: The race of “soft” against “hard”. International Journal of Pharmaceutics.
